# Supplementary material for: Tree-Based Co-Clustering Identifies Chromatin Accessibility Patterns Associated With Hematopoietic Lineage Structure
Source: Front Genet. 2021 Oct 1;12:707117. doi: 10.3389/fgene.2021.707117 (PMC8517275; doi:10.3389/fgene.2021.707117)
Supplement: Supplementary file 1 [file DataSheet1.PDF]

Supplementary Material

1 SUPPLEMENTARY DATA

1.1 Figures

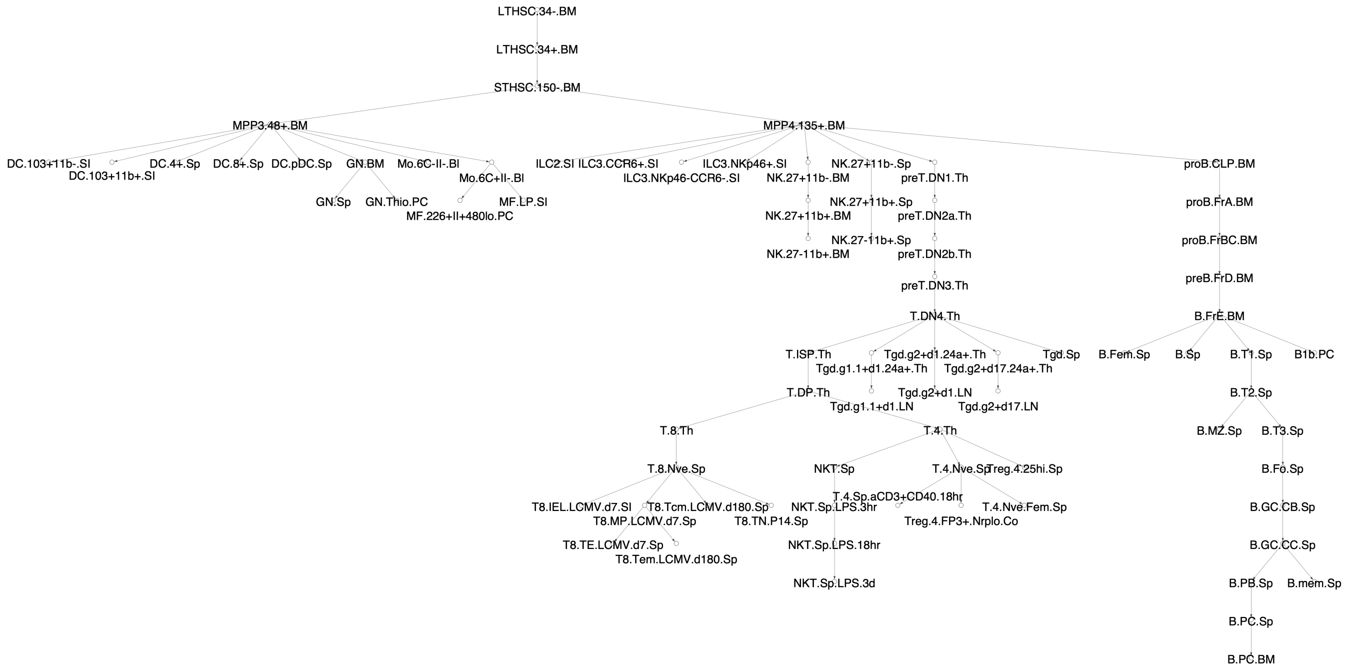

Figure S1. Differentiation Tree. We consider 78 hematopoietic cell types and assume the shown tree structure that exactly matches that of Yoshida et al. in their Figure 1A. In the main text, cell type names are shortened for readability. Here, the full cell type names, corresponding to those of Yoshida et al., are shown
